# Shifting Heights? A 40‐Year Resurvey of Alpine Marmot Distribution in Response to Climate Change

**DOI:** 10.1002/ece3.71777

**Published:** 2025-07-20

**Authors:** Miriam Simma, Arpat Ozgul, Francois Duchenne, Guido Ackermann, Hannes Jenny, Juerg Paul Müller, Anne Kempel

**Affiliations:** ^1^ WSL Institute for Snow and Avalanche Research SLF Davos Switzerland; ^2^ Climate Change Extremes and Natural Hazards in Alpine Regions Research Centre CERC Davos Switzerland; ^3^ Department of Evolutionary Biology and Environmental Studies University of Zürich Zürich Switzerland; ^4^ Swiss Federal Institute for Forest Snow and Landscape Research (WSL) Birmensdorf Switzerland; ^5^ MaB Davos, Project ‘Wildlife’, EAFV/WSL und Bündner Naturmuseum Chur Switzerland; ^6^ Amt Für Jagd und Fischerei Kanton Graubünden Switzerland

**Keywords:** alpine marmot, climate change, dynamic site‐occupancy models, elevational gradient, elevational optimum, range margins, range shift

## Abstract

Alpine species are severely affected by climate change, with elevational range shifts being one key response of mountain species to the rapidly warming environment. The Alpine marmot ( *Marmota marmota* ) is suggested to be particularly susceptible to ongoing warming. However, it is largely unknown how climate change affected the Alpine marmot distribution in recent decades. This study examines the elevational changes in Alpine marmot distribution over the past 40 years in a Central Alps Mountain valley. Based on historical occurrence data of the year 1982, we resurveyed the marmot occurrences in the year 2022. We analysed potential distributional changes over time by fitting dynamic site‐occupancy models to detect occupancy patterns, as well as marmot colonisations and site abandonments (‘local extinctions’ at a site) along the elevational gradient, whilst accounting for imperfect detection. Contrary to expectations, we found no evidence of upward colonisation at higher elevations or an upward shift of the lower range margin in our study, suggesting that marmots are not climate‐limited at lower elevations in the investigated valley, and other factors than climate might constrain their higher elevation colonisation. Nevertheless, the marmot's elevational optimum shifted upwards by +86 m. Our results indicate that the most favourable conditions for marmots have slightly shifted higher due to warming. To better understand potential habitat contractions driven by climate change, further large‐scale studies focusing on the lower range margins in warmer Alpine regions are necessary. Recognising distribution changes of species vulnerable to climate change is crucial to evaluate local extinction risks and for conserving biodiversity.

## Graphical abstract

The Alpine marmot ( *Marmota marmota* ) is an alpine specialist that might be particularly affected by ongoing warming. Based on historical occurrence data from the year 1982, we resurveyed the marmot occurrences in the year 2022 and show that the marmot's elevational optimum shifted upwards by +86 m. In contrast, we found no evidence of upward colonisation at higher elevations or an upward shift of the lower range margin, suggesting that marmots are not climate‐limited at lower elevations, and other factors might constrain their higher elevation colonisation.

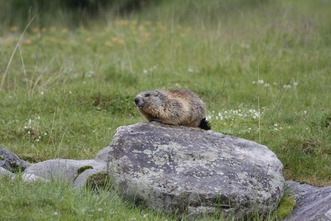

## 1 Introduction

Ongoing climate change is suggested to have tremendous effects on all levels of biodiversity on Earth (Bellard et al. 2012; Walther et al. 2002; Wiens 2016). Alpine ecosystems are expected to be particularly affected by warming (La Sorte and Jetz 2010; Steinbauer et al. 2018), with climate change altering seasonal precipitation patterns, relative humidity and increasing precipitation extremes alongside rising temperatures (Gobiet et al. 2014). These abiotic changes, combined with advancing tree lines, may threaten high‐altitude species, which are adapted to harsh environments with short growing periods and long winters (Dirnböck et al. 2011; Gunderson et al. 2012; Inouye et al. 2000). This is of major concern, as mountain ecosystems are recognised as ‘biodiversity hot spots’ and often host a considerable number of endemic species (Noroozi et al. 2018).

Temperature is a crucial factor limiting species occurrences along elevational gradients (Körner 2007) and several studies report elevational range shifts of various species due to climate warming (Lenoir et al. 2020; Parmesan and Yohe 2003). In the European Alps, the average elevational shift in maximum abundance (elevational optimum) for many species, including plants, animals and fungi, was between +18 and +25 m per decade since 1970. The upper range limits shifted between +47 and +91 m per decade in animal species and between +17 and +40 m in several plant species (reviewed in Vitasse et al. 2021). However, studies on mammalian range shifts in the Alps are rare. Büntgen et al. (2017) analysed the trends of the mean harvest elevation of ungulates between 1991 and 2013 in the Swiss Alps and found a significant increase of harvest elevation for Red Deer (+79.35 m), Chamois (+94.61 m) and Alpine Ibex (+134.86 m) during that period. In another study in the same area, Schai‐Braun et al. (2021) concluded that alpine habitat specialists might respond more slowly to climate change in terms of migration compared to generalists, as specialists could be more at risk of losing suitable habitats over time along the elevational gradient. Thus, the distributional responses of alpine‐obligate species need to be specifically addressed, as these species are important elements of mountain biodiversity.

A typical cold‐adapted specialist in the Alps is the Alpine marmot ( *Marmota marmota* ), a ground‐dwelling sciurid rodent. The Alpine marmot is particularly sensitive to high temperatures and highly territorial (Arnold 1999). Marmots are well adapted to cold conditions through social hibernation but are limited by high temperatures during the growing period, which affects their ability to accumulate body fat necessary for winter survival (Türk and Arnold 1988). Alpine marmots live in complex family groups led by a dominant breeding pair and their subordinate offspring. Mutual warming within these groups during hibernation (October–April) is crucial, especially for juveniles (Arnold 1999). During their short active season, they must rapidly increase their weight by 30% in adults and 180% in pups to sustain hibernation (Signorell and Jenny 2003). Climate change impacts on marmots include reduced snowpack insulation, affecting winter energy expenditure and survival rates (Rézouki et al. 2016; Tafani et al. 2013). Due to their low genetic diversity caused by historical climate shifts, current rapid warming may exceed their adaptive capacity (Gossmann et al. 2019). Despite variations between species and regions, Armitage (2013) suggested that marmots are unlikely to survive long‐term in a warm, dry climate. Notably, the Alpine marmot exhibits the lowest genetic diversity among wild animals, likely due to repeated climate shifts during the Pleistocene and extreme niche adaptation (Gossmann et al. 2019). Although it has persisted with low genetic variation, this is expected to hinder their adaptation to rapidly warming climates. Predicted habitat loss for Alpine marmots, even under modest climate change scenarios, is concerning (Glad and Mallard 2022). The upward shift of treelines in the Alps is already reducing suitable habitats (Bebi et al. 2017; Gehrig‐Fasel et al. 2007). With limited genetic and phenotypic adaptability (Rézouki et al. 2016; Tafani et al. 2013), marmots may need to migrate to higher elevations to survive (Gunderson et al. 2012). In the Alps, marmots are typically found between 1200 and 2700 m asl., but up to 3000 m asl., avoiding the forest belt and favouring grasslands and microclimates with specific slopes and vegetation (Allainé et al. 1994; Arnold 1999; Borgo 2003; Galluzzi et al. 2017). Elevation is a key factor in their distribution (Glad and Mallard 2022), suggesting that climate change‐driven migration should be observable along elevational gradients.

In this study, we took advantage of historical marmot occurrence data collected in 1982 in the Dischma Valley, Swiss Alps (Müller et al. 1988) and resurveyed the marmot occurrences in the same area in 2022. During the last decades, temperature rises in Switzerland were twice as high compared to average temperature trends in the Northern Hemisphere, exceeding an increase of 2°C (FOEN Switzerland 2020; Rebetez and Reinhard 2008). Moreover, since the 1970s, the mean annual maximum snow depth, the snow cover duration and the number of days with snow on the ground have declined across the Swiss Alps (Klein et al. 2016). The aim of this study was to assess whether the Alpine marmot has colonised higher elevations in response to the rapid environmental changes during the last four decades. For this purpose, we used dynamic site‐occupancy models based on the occurrence data from the two timepoints (1982 and 2022) to analyse how elevation, slope, aspect and habitat type affected the initial marmot site‐occupancies in 1982 and to estimate colonisation and local extinction rates along the elevational gradient over time whilst accounting for imperfect detection (MacKenzie et al. 2003). To enable a better understanding of the climatic conditions and the magnitude of climate warming in the study area, we investigated trends of climatic parameters specifically relevant for marmots measured at a nearby meteorological station. We expected to find evidence of climate warming and an upward movement of the marmots' range, and hence, a colonisation of higher elevations over time. As microclimate is important for marmots and migration could be restricted by a lack of habitat suitability at higher elevations, we assumed a change in aspect preference deviating from south to be another possible response to climate change. Investigating these aspects of site‐occupancy dynamics can shed light on climatically driven distribution changes of the Alpine marmot.

## 2 Material and Methods

### 2.1 Study Area

**FIGURE 1:**
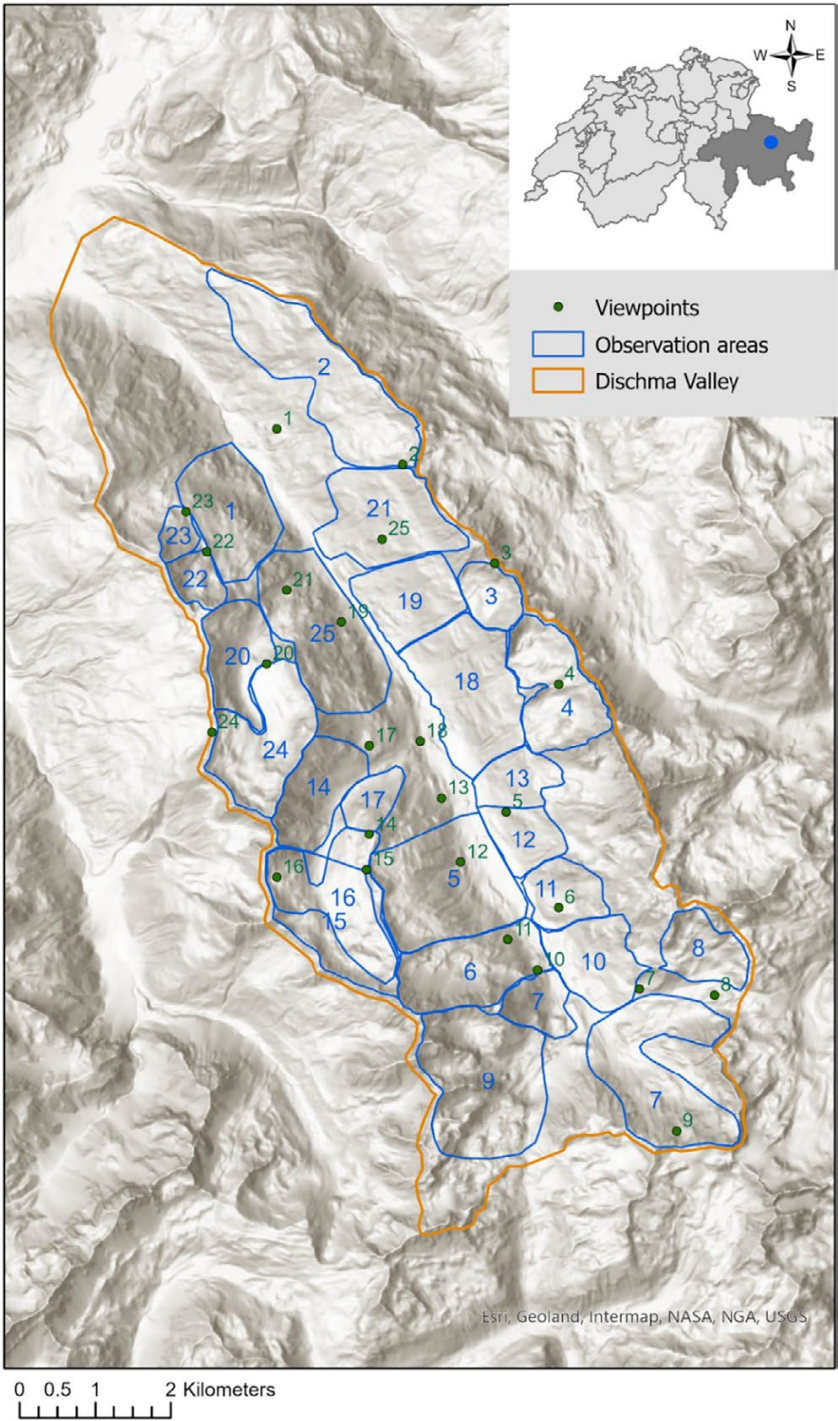
Observation areas and corresponding viewpoints in the Dischma Valley, canton Grisons, Switzerland. The size of the polygons varied between 27 ha (Nr. 23) and 280 ha (Nr. 9).

The study was conducted in the Dischma Valley, a mountain valley in Davos, Switzerland.

(Figure 1). The valley is 55 km^2^ in size and ranges from 1550 to 3130 m asl. It runs NNW–SSE, and therefore, the two downhill sides mainly face southwest and northeast, respectively. Belonging to the Western Central Alps, the area is characterised by a temperate central alpine climate. The mean annual temperature measured at the meteorological station in Davos was 3.33°C in 1982 and 5.30°C in 2022 (DAV, 1594 m asl; geographic coordinates: 46.812969/9.843558; MeteoSwiss 2022). The spatial meso‐climatic differences within the elevational range of the study area allow for a variety of habitats, including closed forest (up to 2100–2200 m asl) (Frei et al. 2023), dwarf‐shrub heath, alpine grassland and subnival rocky habitats (Landolt et al. 1986). Grassland management is a major factor that shapes the landscape of the valley and maintains open habitat structures independent from the potential naturally occurring vegetation type. Whilst regular mowing is restricted to the valley bottom, extensive grazing by livestock takes place throughout large parts of the grassland habitats in the valley (Landolt et al. 1986). The hunting season for marmots in the area is annually restricted to a period of 3 weeks in September (Canton Grisons 2022).

### 2.2 Field Sampling in 1982 and 2022

In a multi‐species vertebrate survey in 1982, the distribution of the Alpine marmot was mapped in the Dischma Valley by recording marmot sightings within 25 defined observation areas across the valley. For this purpose, the researchers visited 25 selected viewpoints, each of them corresponding to a certain observation area (Figure 1), between July and September (Müller et al. 1986). Binoculars and telescopes were used to detect marmot individuals over large distances, which were up to 3602 m, from the viewpoint to the edge of the observation area (Figures S1 and S2). The survey durations at a single viewpoint ranged mainly between 1 and 2 h, and viewpoints were mostly visited twice during the historical sampling. As ungulates were also recorded, most surveys were either conducted in the early morning or in the evening. The surveyed observation areas covered an area of 37.4 km^2^ and overall, they ranged from 1656 up to 3093 m asl. To ensure comprehensive coverage of species occurrences, observers also conducted additional, unsystematic observation tours on foot. However, sightings from these tours were excluded from the present study to maintain comparability between the historic and recent surveys.

In July and August 2022, we revisited the 25 viewpoints and resurveyed the same observation areas for marmot occurrences. We recorded the location of sighted marmots on a digital orthophoto using the ArcGIS Survey 123 data collection app. To collect data comparable to the historical sampling design, we visited each viewpoint twice and surveyed the same areas for 1–2 h. For each area, we conducted one survey in the first half of the day and one survey in the second half of the day. Midday heat was avoided, and no surveys were conducted on rainy days, as marmots are unlikely to appear above ground under those two weather conditions (Türk and Arnold 1988). The unsystematic surveys carried out by foot in the past were not replicable, and hence, the resurvey in 2022 was restricted to the viewpoint visits.

### 2.3 Temperature and Precipitation Changes

To demonstrate the magnitude of climate change in Davos during the last decades, we used the temperature and precipitation data measured at the meteorological station in Davos (DAV, 1594 m asl; geographic coordinates: 46.812969/9.843558; MeteoSwiss 2022) in the period from 1961 to 2022. Alpine marmots are sensitive to high temperatures during the activity period, and winter survival is particularly affected by the thickness of the snowpack (Rézouki et al. 2016; Türk and Arnold 1988). Thus, climatic trends were assessed on a seasonal level. We illustrated the mean temperature and total precipitation in the marmot activity period (April—September, hereafter referred to as ‘warm season’) and the hibernation period (October–April, hereafter referred to as ‘cold season’) of each year. Additionally, from the measured daily temperature maxima, we derived the number of days per year at which temperatures ≥ 25°C were measured.

### 2.4 Data Preparation for Occupancy Analysis

The historical maps of the marmot records were georeferenced and digitised in 2022. We excluded marmot presence points that were, according to the notes on the historical maps, indicated as being recorded during a tour by foot in the historical data to obtain comparable data with the ones from 2022.

#### 2.4.1 Detection History

The observation areas ranged between 27 and 280 ha in size. To define ‘sites’ as spatial units with potential marmot occupancy for the analysis, we subdivided the surveyed areas into 100 × 100 m grid cells, resulting in 3426 sites. Based on the point data of marmot detections in each survey (i.e., a sampling event occurring at time *t* at site *i*), we derived a detection history for each 100 × 100 m site. A detection history for dynamic site‐occupancy models consists of 1 or 0 (detection/non‐detection) for each site *i* and survey *k* (MacKenzie et al. 2003). Each observation area was surveyed twice in 2022; however, no survey‐specific information (number of viewpoint visit, survey date) was available for the marmot presence points in 1982. Therefore, we considered the marmot presences in 1982 collectively as data from one (primary) survey, so there was no second survey to assess the detection probability. To account for imperfect detection in the past, the detection probability in the historical survey was assumed to be the same as—and thus estimated from—the repeated survey of 2022.

#### 2.4.2 Environmental Variables and Survey Distance

We obtained the environmental covariates elevation, slope and aspect (i.e., the exposition of the slope) of each 100 × 100 m site from the SwissAlti3D digital elevation model, which is provided by the Swiss Federal Office of Topography. As elevation was available in a 0.5 × 0.5 m resolution and aspect and slope in a 2 × 2 m resolution, we aggregated the values to a 100 × 100 m resolution by using the mean of the aggregated grid cells. Vegetation data of 1982 were available from precise vegetation mapping during the historical sampling period (Müller et al. 1986) in a 50 × 50 m resolution. We categorised the detailed vegetation maps into five habitat categories (Table 1) and aggregated them to a 100 × 100 m resolution to obtain the dominating habitat category for each site. To include the slope aspect in the model, we computed the aspect cosine in radians, measured clockwise from north (Vandegehuchte et al. 2017). The derived index is a continuous variable termed ‘northness’.

**TABLE 1:** Defined habitat categories derived from the vegetation maps of the Dischma Valley in 1982.

Habitat category	Description
Forest	Forests and subalpine krummholz
Dwarf‐shrubs	Shrublands predominated by dwarf‐shrubs (mainly family Ericaceae)
Grassland	Alpine grasslands, meadows, pastures and snow beds
Scree	Rocky habitats with sparse vegetation, predominated by silicate scree
Other	Silicate bare rock, water bodies, firn, residential areas

To account for imperfect detection related to large survey distances, we calculated the linear distance (m) between each site (grid cell) and its corresponding viewpoint and used it as a covariate for detection probability in the occupancy models. Two observation areas (Nr. 15 and 16) overlapped (see Figure 1) and large parts of them were visible and surveyed from two different viewpoints (Nr. 15 and 16). Thus, we calculated the distances from each overlapping grid cell to both corresponding viewpoints and considered the mean of the two distance values as the survey distance of those sites.

The digitisation of the historical data, the preparation of the marmot point data and the raster datasets of the environmental covariates (elevation, aspect, slope, habitat category) was conducted in ArcGIS Pro version 3.0.3 by using the extensions ‘3D Analyst’ and ‘Spatial Analyst’. Further data preparation, in particular the preparation of the detection history for the occupancy modelling and the calculation of the survey distances for each site was done in the R programming software (R Core Team 2021).

### 2.5 Dynamic Site‐Occupancy Models

To analyse the marmots' distribution and potential colonisation or local extinction processes along the elevational gradient over time, we used the dynamic site‐occupancy model of MacKenzie et al. (2003). Dynamic site‐occupancy models were developed to model the site‐occupancy probability of species and the changes in occupancy states over time driven by colonisation and local extinction, whilst accounting for imperfect detection. For that purpose, the model considers and links two processes occurring during the field sampling: occupancy and detectability. The occupancy represents the ‘true’ state of presence or absence of the target species at the given sites during a sampling period. Since we assumed that detectability was not perfect, this ‘true’ state of presence/absence was never observed directly. Detectability accounts for non‐detection during a survey whilst the species was present (false negative). Detectability can vary with climatic conditions, because it affects species behaviour for example, or because of observation conditions (e.g., survey distance, vegetation properties). To estimate detection probability, at least two surveys are required within each sampling period. One important assumption of the model is that the state of occupancy at a site can change between periods (historical sampling and resurvey), but not between surveys of a period. Therefore, the two surveys are repeated visits to estimate the detection probability (MacKenzie et al. 2003). The four estimated parameters of the model are:

1. The *detection probability* (*p*_*i*_), which is the probability of detecting the target species at site *i* in case of its presence.
2. The *initial occupancy probability* (ψ_*i*_), which is the probability of species occurrence at site *i* in the historical survey (*t* = 1, in this study in 1982).
3. The *colonisation probability* (γ_*i,t*_), which is the probability of a previously unoccupied site *i* being colonised during the time period *t* and *t* + 1, in this study between 1982 and 2022.
4. The *local extinction probability* (ε_*i,t*_), which is the probability of a previously occupied site *i* becoming unoccupied during the time period *t* and *t* + 1, in this study between 1982 and 2022.

The details of the parameters' estimation (e.g., calculation of likelihood) have been described in MacKenzie et al. (2003).

### 2.6 Implementation of the Occupancy Model

We performed the occupancy modelling in the R programming software (R Core Team 2021) by using the package ‘unmarked’ (Fiske et al. 2015). We used the marmot detections/non‐detections at each site *i* and survey *k* as the binary response variable *y*_ikt_ and fitted a set of hypothesis‐driven candidate models by using the site‐covariates elevation, slope, northness and habitat category as predictors for the occupancy state, elevation and northness as hypothesised drivers of the colonisation and extinction process and distance from the viewpoint to each site as site‐covariate for the detection process. As the detection history included only one survey for 1982, we had to assume that the detection probability in 1982 was similar to the one estimated from the repeated survey in 2022. Whilst this is not ideal, this approach seems a reasonable assumption given that landscape characteristics and vegetation have not changed in a way that would bias detectability, particularly not in relation to elevation. We compared the models using AIC (Akaike information criterion) and selected the most parsimonious model (with the lowest number of parameters) out of the best‐approximating models (ΔAIC < 2; Bruggeman et al. 2016). To assess model fit, we applied the Mackenzie‐Bailey Goodness‐of‐Fit test (*n* simulations = 2000) and derived the *č* value (variance inflation factor). A *č* value around 1 suggests that the model fits the data adequately, whereas a value greater than 1 indicates overdispersion (variation in the data is larger than expected by the model) and a value smaller than 1 indicates underdispersion (variation in the data is smaller than expected by the model; MacKenzie and Bailey 2004). We applied non‐parametric bootstrapping implemented in the ‘nonpaarboot’ function of the ‘unmarked’ package to calculate the occupancy estimates of the two survey periods (bootstrap samples per season = 100). We derived the predicted values of the covariates for each parameter of the model using the ‘unmarked’ package.

To better understand the colonisation and extinction processes, we additionally illustrated the occupancy distribution in 2022, calculated from the predicted values of the model by using the following equation:

$$\Psi_{i,t=2022}=\Psi_{i,t=1982}\left(1-\varepsilon_{i}\right)+\left(1-\Psi_{i,t=1982}\right)\gamma_{i}$$

95% confidence interval associated with this prediction was calculated using bootstrapping values in the confidence interval of each parameter.

To assess potential shifts in the elevational range between 1982 and 2022, we used the predicted values to calculate the average elevation of occupied sites as well as the elevational maximum of site‐occupancy in both years. The ‘maximum probability of species occurrence’, considered as potential ‘optimum’, is an informative additional measurement to assess species distribution responses to large‐scale environmental change (Lenoir et al. 2008; Moritz et al. 2008).

## 3 Results

### 3.1 Local Temperature and Precipitation Trends

**FIGURE 2:**
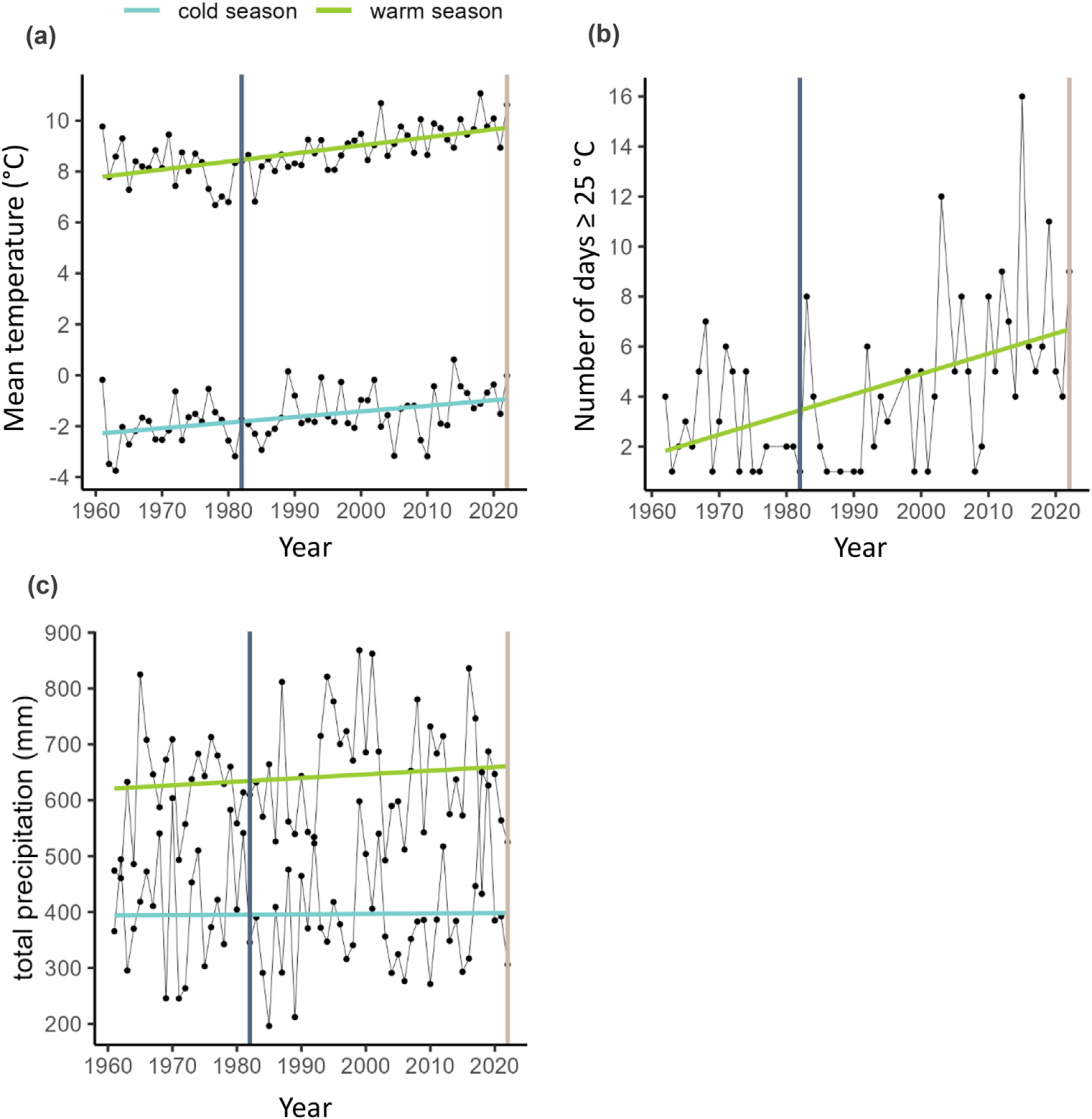
Trends in mean temperature (a), number of days with temperature maxima ≥ 25°C (b) and total precipitation (c) per year in Davos between 1961 and 2022. The ‘cold season’ includes measurements from October to April (hibernation period), the ‘warm season’ includes measurements from April to September (growing period); black points and lines represent raw data; vertical lines denote the year of the historical survey (1982) and the resurvey (2022). Note that the actual values are not representative for the higher situated study area, but trends in Davos might be indicative for trends in the nearby Dischma Valley.

Since the marmot surveys in 1982, the mean temperature trends in Davos show an increase of 1.04°C over 40 years (estimate for year = 0.026 ± 0.004, *p* < 0.0001) in both the warm season (estimate for year = 0.032 ± 0.005, *p* < 0.0001), during which marmots are active, and the cold season (estimate for year = 0.022 ± 0.006, *p* = 0.007), during which marmots hibernate (Figure 2a). The number of days with temperature maxima ≥ 25°C was one in 1982 and nine in 2022 and increased significantly over the years (estimate for year = 0.17383 ± 0.053, *p* = 0.0027). Hot days occurred more frequently during the last two decades (Figure 2b). In contrast, changes in total precipitation showed no general pattern (estimate for Year = 0.117 ± 0.17, *p* = 0.494, Figure 2c).

### 3.2 Occupancy Analysis

In all surveys in 1982, marmots were detected at 117 different sites. In 2022, we detected marmots at 37 sites in the primary survey and at 42 sites in the secondary survey. Repeated detections in the two surveys in 2022 occurred at 9 sites, which results in 70 sites that included at least one marmot detection during that year (i.e., 40% less sites with a detection relative to 1982). Only at 8 sites were marmots detected at least once in both years (1982 and 2022).

The model selection revealed four best‐approximating models (ΔAIC < 2; Table S1). Out of those, the most parsimonious model (model with the lowest number of parameters) included distance from the viewpoint to the observational site as a covariate for the detectability, elevation as linear and quadratic covariates for the colonisation, extinction and initial state occupancy, and northness and habitat category as covariates for the initial state occupancy.

The Goodness‐of‐Fit test assessed for the selected model (bootstrap samples *n* = 2000) indicated slight but non‐significant underdispersion (*p* = 0.491; *č* = 0.84; Figure S3). The estimated mean smoothed occupancy probability per site was 0.32 (SE ±0.17) in 1982 and 0.10 (SE ±0.05) in 2022. This apparent decline in average occupancy over time must be treated with caution because of the heterogeneity in sampling intensity between 1982 and 2022, and additionally because of the consideration of the historical sampling events as one collective survey in the occupancy model.

**FIGURE 3:**
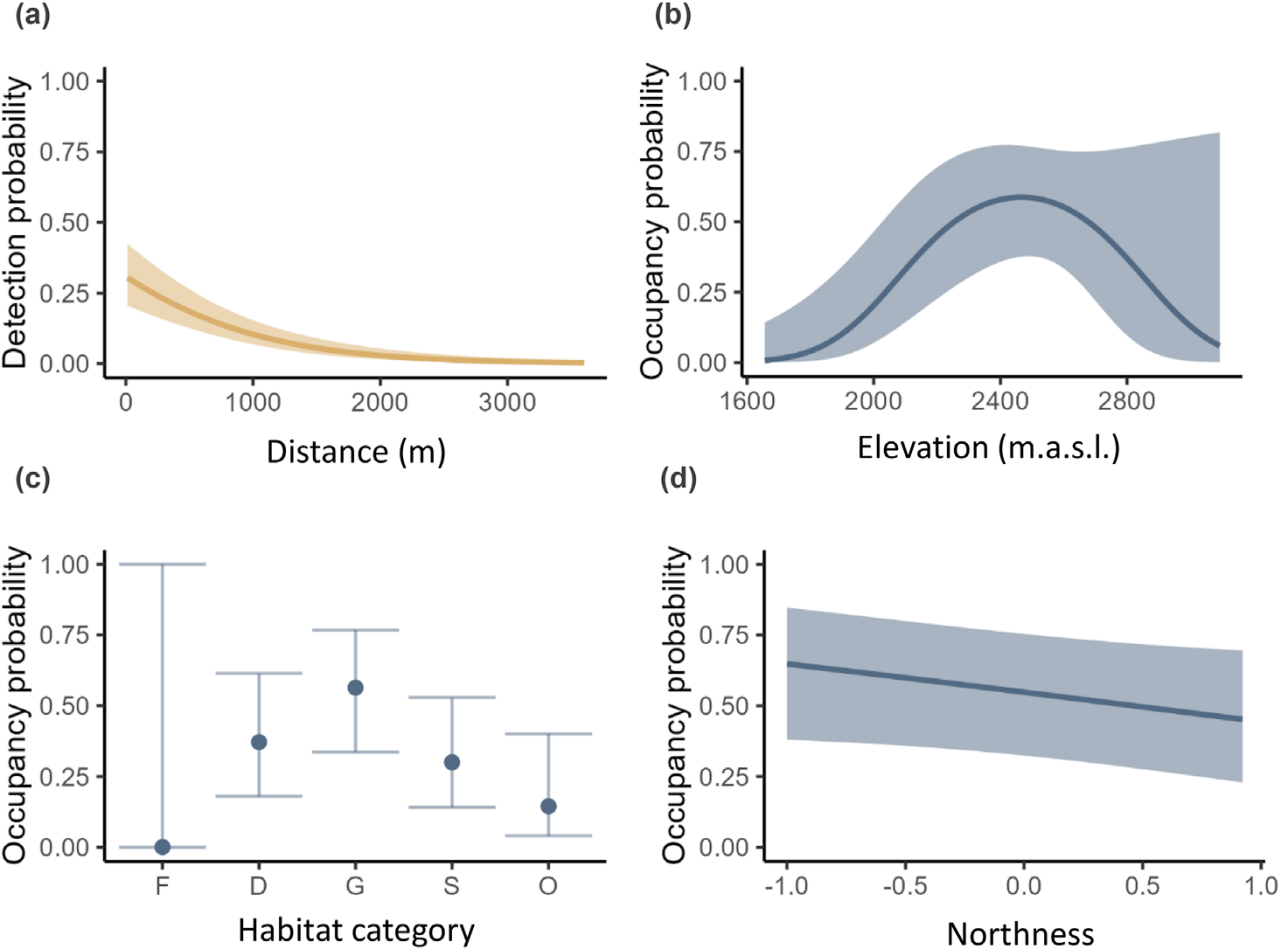
Estimated detection probability in relation to survey distance (a) and estimated initial occupancy probability (in 1982) in relation to elevation (b), habitat category (F = forest, D = dwarf‐shrubs, G = grassland, S = scree, O = other) (c) and northness (with 1 indicating the highest northness) (d). Lines are predicted values from the dynamic site‐occupancy model. Predictions of elevation were done for the grassland habitat and average northness value. Predictions of northness were done for the grassland habitat and average elevation value. Shaded areas and error bars represent 95% confidence intervals. Forest sites were underrepresented in the data (see Figure S4), causing a large CI.

Elevation significantly affected the initial state occupancy in 1982 in a non‐linear way (Table 2, Figure 3b), defining the historical elevational range of the marmot population. Site‐occupancy peaked around 2500 m asl, whilst elevations below 2000 and above 2900 m asl were unlikely to be occupied in the past. The marmots' preference for grassland habitats, a lower possibility of occurrence in scree and dwarf‐shrub dominated habitats, and its natural absence in forest habitats was indicated but not significant (Table 2, Figure 3c). Similarly, the negative relationship between northness and site‐occupancy in 1982, which indicates that marmots tend to prefer south‐, southeast‐, or southwest‐facing slopes, was not significant (Table 2, Figure 3d). According to the model selection, slope was a dispensable site‐specific covariate and, thus, of minor relevance for the site‐occupancy states (see Table S1). The detection probability derived from the repeated surveys in 2022 was low and decreased considerably with survey distance (Table 2, Figure 3a). Marmots were very unlikely to be detected from a distance > 1500 m.

**TABLE 2:** Parameter estimates of the model selected on the basis of parsimony.

Initial state occupancy probability	Estimates	SE	*z*	*p*
Intercept	−6.89	24.65	−0.28	Marginal
Elevation	0.46	0.31	1.52	0.130
Elevation^2^	−0.55	0.25	−2.21	**0.027**
Northness	−0.21	0.14	−1.53	0.125
Dwarf‐shrubs	6.36	24.65	0.26	0.796
Grassland	7.15	24.65	0.29	0.772
Scree	6.04	24.65	0.25	0.806
Other	5.11	24.66	0.21	0.836
Colonisation probability				
Intercept	−17.08	7.26	−2.35	Marginal
Elevation	−19.93	9.84	−2.03	**0.043**
Elevation^2^	−6.45	3.28	−1.97	**0.049**
Local extinction probability				
Intercept	1.00	0.30	3.34	Marginal
Elevation	−0.51	0.54	−0.96	0.340
Elevation^2^	0.19	0.74	0.25	0.802
Detection probability				
Intercept	−2.48	0.24	−10.20	Marginal
Distance	−0.93	0.13	−7.20	**< 0.0001**

*Note:* Estimates are on logit scale. Predictor variables are z‐transformed. Forest is the reference level in the intercept of the initial state occupancy regression. *p*‐values considered significant are highlighted in bold.

**FIGURE 4:**
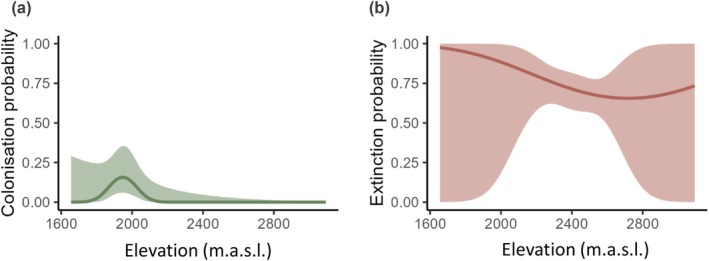
Estimated colonisation probability (a) and local extinction probability (b) in relation to elevation. Lines are predicted values from the dynamic site‐occupancy model. Shaded areas represent 95% confidence intervals.

**FIGURE 5:**
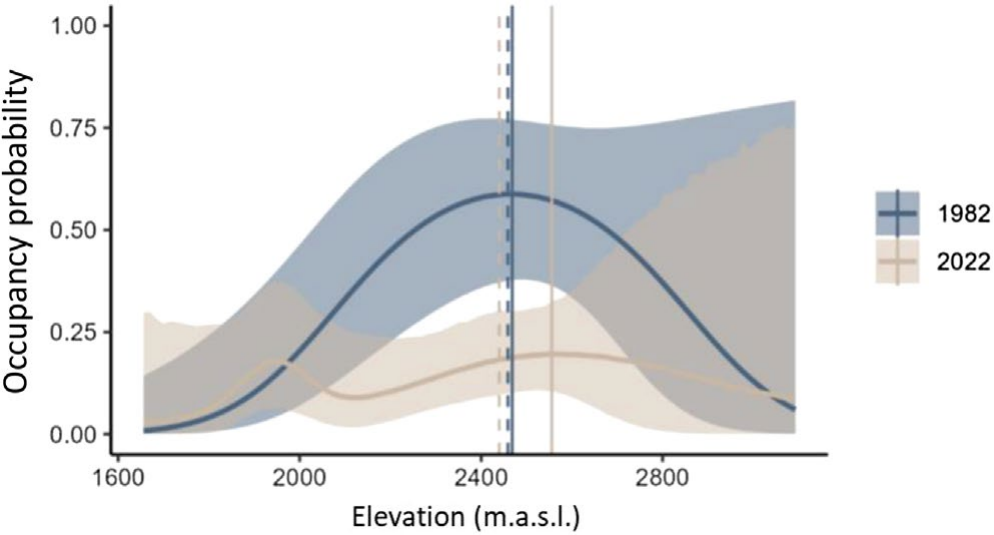
Estimated occupancy probability in 1982 and 2022 in relation to elevation. The occupancy distribution in 1982 is represented by predicted values from the dynamic site‐occupancy model; the distribution in 2022 was estimated by using ψ_2022_ = ψ_1982_ × (1‐ε_i,1982_) + (1‐ ψ_i,1982_) × y_i,1982_. Shaded area represents 95% confidence intervals; solid lines = maximum of occupancy probability; dashed lines = mean elevation of occupancy. Note that the comparability of the absolute occupancy values between the years is widely limited.

The colonisation probability was highest at the lower part of the historical elevation range, between 1900 and 2000 m asl, whereas higher elevations were unlikely to be colonised over the 40‐year period from 1982 to 2022 (Table 2, Figure 4a). Likewise, the local extinction probability tended to be higher at low elevations, below 2000 m asl, but this trend was not significant and included a considerable degree of uncertainty (Table 2, Figure 4b). Moreover, northness played a negligible role in the process of colonisations and local extinctions, indicating that colonised sites at present do not differ significantly in slope aspect relative to the sites occupied in the past (see Table S1). The balance between colonisation and extinction rates led to a bimodal occupancy distribution along the elevational gradient in 2022 (Figure 5). The present distribution indicates a slight decrease in average elevation of occupied sites (1982: 2459 mL; 2022: 2429 m) and an upward shift by +86 m of the maximum probability of occupancy (1982: 2469 m; 2022: 2555 m), the elevational ‘optimum’, during the 40‐year period.

## 4 Discussion

Our study on range dynamics of the Alpine marmot in an era of rapid climate change shows that over the last four decades, we detected increased colonisation at low elevation but no colonisation of sites at higher elevations in our study area. This indicates that no upward shift of the marmots' upper and lower range margins in our study region was detected, likely because climate is still not limiting at lower elevation in the observed area, and other factors constrained marmots at high elevation. However, when observing the marmot's elevational optimum, we found an increase of +86 m, indicating an overall upward movement of the marmot in response to warming. We discuss our findings and potential caveats of our study in the context of our expectations to better understand the range dynamics of the Alpine marmot in a changing world.

### 4.1 No Changes in Marmot's Upper and Lower Range Margins but Slight Increases in Marmot's Elevational Optimum

Despite climate warming and in contrast to our expectations, our observations reveal no colonisation process at higher elevations by marmots. The absence of colonisation at higher elevations over time suggests a constraint on the upward shift of the marmots' upper range margin within our study region, the Dischma Valley. Notably, marmots were already established on ridge sites across most observation areas as early as 1982 (Müller et al. 1986). Due to the topographical constraints of the mountains, migration possibilities upwards over the last four decades have therefore been restricted to a few areas at the back of the valley. Whilst this may raise concerns regarding long‐term extinction risks, it has significantly limited the probability of detecting high‐altitude colonisation within the timeframe of our study. Where topographical constraints are less relevant, the lagging of soil development behind climate warming is assumably a crucial factor that severely limits colonisations of higher altitudes by the Alpine marmot (Rehm and Feeley 2016; Seastedt and Oldfather 2021). The presence of soil conducive to burrowing and resistant to freezing remains a vital habitat attribute essential for the marmots throughout the year and under a range of climatic conditions.

Similarly, no discernible upward shift of the lower range margin was observed. Rather, marmots exhibited a slight extension of their elevational range downwards, resulting in a marginally lower average elevation of occupied sites compared to 1982. This suggests that lower sites within the valley remained climatically suitable for marmots. Analysis of temperature data from a nearby meteorological station at 1594 m asl revealed that the number of days with temperatures ≥ 25°C per year typically ranged between 3 and 6 days. Consequently, instances of heat stress among marmots were infrequent in the Dischma Valley over recent decades, implying that temperature alone likely had no direct adverse impact on marmot persistence at lower elevations in the surveyed areas. Previous studies have demonstrated negative effects on marmot foraging and aboveground activities only when ambient temperatures exceed 25°C (Türk and Arnold 1988). Therefore, it is plausible that such unsuitable temperature conditions would need to occur more frequently within and across summer seasons at occupied sites to induce a retreat of the marmots' lower range margin in the Dischma Valley.

Other factors beyond elevation had only weak effects on the occupancy probability of marmots. Whilst we observed a slight preference for grassland habitats and south‐facing slopes, these effects were not statistically significant. Nevertheless, the latter in particular suggests that marmots may still be able to respond to increasing heat stress by shifting towards slopes with more northern inclinations. This mechanism—using fine‐scale topographic variation to buffer against climate warming—has been well documented in alpine plants, where microclimatic heterogeneity enables persistence despite broader climatic trends (Jiménez‐Alfaro et al. 2024; Opedal et al. 2015; Scherrer and Körner 2011). Such fine‐scale shifts in slope aspect may help marmots maintain suitable thermal conditions and support their resilience under future warming.

Whilst our study did not reveal significant changes in the marmot's range margins, we did observe a notable shift in the maximum probability of marmot occurrence, approximately 86 m higher compared to 1982. This shift in maximum probabilities offers an alternative indicator of species responses to climate change, complementing assessments of range margins. Focusing solely on range margins may overlook crucial distributional dynamics, as extremes can be influenced by individuals temporarily venturing beyond their fundamental niche (Tingley and Beissinger 2009). This shift aligns with trends found for more mobile and generalist mammals in Grisons (see Büntgen et al. 2017), supporting the expectation that the Alpine marmot responds to ongoing climate change through upward migration.

### 4.2 Changes in Marmot's Occupancy Along the Elevational Gradient

The elevational distribution in 1982 shows a clear peak in marmot's maximum occupancy probability at mid‐elevations, whereas the occupancy distribution in 2022 is bimodal, with high occupancy probabilities both below and above the forest line (2100–2200 m asl in the study area, Frei et al. 2023). The change in the elevational distribution is also indicated by increased colonisation events at low elevation in the recent survey. The observed bimodal distribution of marmots in the Dischma Valley appears logical, given their preference for open habitats, which we also found in our study (week tendency for grasslands). Anthropogenic land‐use practices, such as mowing, fertilisation and temporal intensive livestock grazing, prevalent in the valley bottom (Müller et al. 1988), seem to benefit marmots by maintaining open habitats and increasing the quality and quantity of herbal food resources. This favourable environment allows marmots to extend their occupancy beyond habitats above the tree line and to expand across a broader elevational range. However, the absence of such a bimodal distribution and an additional occurrence peak at low elevation in the past suggests potential changes in land use or human behaviour over time at lower elevations.

For instance, marmots inhabiting intensively managed grassland habitats at the valley bottom have historically been perceived as pests by farmers, leading to targeted hunting in these areas (Müller et al. 1986). Relaxed hunting pressure at lower elevations could explain the observed increase in marmot colonisation, and indeed, numbers of hunted marmots in the region of Davos declined by approx. 20% over the last 30 years (Figure S5). However, personal communication with farmers from the valley suggests that hunting pressure has been negligible for decades, questioning the potential significance of hunting impacts on marmot occurrences in our study. Similarly, shifts in the natural predator populations of marmots, such as red foxes ( *Vulpes vulpes* ) or golden eagles ( *Aquila chrysaetos* ) may have influenced their spatial distribution. Whilst we lack information on the development of the red fox population, predation by golden eagles is likely to have been constant, as there is currently and has been one breeding pair in the study area. However, the impact of predation on marmot colony survival is expected to be minimal compared to the effects of winter severity (Zeiler and Preleuthner 2015). Consequently, relaxed hunting pressure might be responsible for the shift in marmot occurrence patterns from a hump‐shaped distribution to a bimodal distribution, although evidence is only weak. Nevertheless, despite the increased colonisations at lower elevations, we still observe an upward shift of marmot's optimum occurrence. This suggests that certain factors influencing marmot's spatial distribution above the treeline have shifted to higher elevations.

### 4.3 Potential Reasons for an Initialised Upward Shift of the Marmot's Elevational Optimum

In the past, marmot site‐occupancy states were clearly influenced by elevation. Several factors could be considered as drivers of the observed relationship. Firstly, most grassland habitats occurred at mid‐elevations (see Figure S6), which may partially explain the marmot occurrence pattern along the elevational gradient. Treeline advances at higher elevations might have occurred and could be responsible for the slight increase in optimum occurrence (Bebi et al. 2017). Secondly, spatial precipitation patterns, particularly snow deposition, could significantly affect marmot distribution due to the importance of snowpack in insulating burrows during hibernation (Rézouki et al. 2016; Tafani et al. 2013). Grünewald et al. (2014) found that in the Dischma Valley, snow depth generally increases with elevation up to around 2400 m before decreasing at higher elevations. This pattern closely resembles the elevational distribution of marmot occupancy. Despite a positive elevational gradient of snowfall, local terrain, wind and gravity lead to snow accumulation on gentle slopes and less snow on steep slopes, typically found at the highest elevations (Grünewald et al. 2014). Thus, spatial snow patterns might provide a missing link to explain the marmot occurrence patterns, and potential changes in snow cover might partly explain occupancy dynamics observed in response to climate change. Third, biotic interactions, such as the occurrence patterns of favoured plant species, could significantly influence the distribution of Alpine marmots and consequently their response to climate change (Freeman et al. 2018). Marmots exhibit selective feeding behaviours on specific plant parts and species (Armitage 1979; Garin et al. 2008). For instance, it has been suggested that marmots may be constrained by plants high in linoleic acid (Ruf and Arnold 2008), which enhance thermoregulation capacities of hibernators by extending torpor bouts and reducing the tolerance limit to cold temperatures (Geiser and Kenagy 1987; Thorp et al. 1994). Climatically induced changes in plant species (e.g., see Mamantov et al. 2021; Rumpf et al. 2018) and shifts in snow patterns along the elevational gradient could potentially impact the range dynamics of marmots. However, delving into these mechanisms was beyond the scope of the current study. Future research focused on investigating how marmots respond to specific food plants or altered snow patterns could provide valuable insights into the underlying mechanisms driving marmot occurrence and potential range shifts. In addition, whilst our study provides fine‐scale insights into the elevational changes in Alpine marmot occupancy, future research could integrate larger‐scale opportunistic datasets (e.g., GBIF or citizen science records) to assess whether the observed patterns are consistent across broader spatial scales.

### 4.4 Challenges Associated With Historical Datasets and Caveats of Our Study

Whilst historical datasets such as the one used in this study represent invaluable treasures, enabling the identification of long‐term trends, patterns and changes in species distributions over time, they also present several challenges. For example, in contrast to the resurvey in 2022, the historical survey had observers focusing not solely on marmots but also on ungulates. Consequently, surveys were mostly conducted early in the morning or in the evening. Moreover, deviations from the usual survey duration of 1–2 h were occasionally possible for unknown reasons. As a result, we were unable to fully account for the sampling heterogeneity between the historical survey and the resurvey. However, we strived to observe marmots under comparable conditions as much as possible. Surveys were restricted to suitable conditions for marmot activity, such as avoiding rainy days. This practice was likely consistent across both time points, as the extensive survey distances made sampling during adverse weather impractical. Furthermore, conducting investigations of the same observation areas from the same viewpoints in 1982 and 2022 suggests that spatially dependent detection probability is similar between the two time points, thus ensuring comparability of the occupancy patterns along environmental gradients.

In addition, the detectability of marmots was very low in our study, which may have substantially reduced the power of the data. Whilst we accounted for the influence of survey distance on the observation process, several other factors, such as daytime, prevailing temperature conditions and disturbance by livestock or humans, might have contributed to an underestimation of ‘false absences’ and thus, to a reduced significance of the data (Dénes et al. 2015). Observers in 1982 knew the area and locations with marmot activity very well, as they performed surveys of other animals in the area already in winter and spring, which might have helped them to locate and find marmots from the distance during their surveys. Moreover, we observed notable differences in site‐occupancy probability between the historical survey and the resurvey, suggesting a potential decline in marmot population size over time. However, whilst occupancy probability is typically estimated from primary surveys and detection probability from secondary surveys within a season (see MacKenzie et al. 2003), historical data collected across multiple surveys were treated as one primary survey due to a lack of detailed information about individual sampling events in 1982. As a result, we estimated site occupancy in 1982 using detection probabilities derived from repeated surveys in 2022. Although this provides a more reasonable estimate of occupancy probability in 1982, it may have led to an overestimation relative to 2022. Consequently, comparing absolute values of occupancy probabilities between the 2 years is inherently limited, and drawing inferences about a population decline over the 40‐year period is unfeasible with our data. Furthermore, marmot population dynamics can vary across space and time due to environmental conditions and demographic factors (Armitage 1991; Boero 1999; Oli and Armitage 2004), making interpretations of population trends based on only two survey years debatable. Nevertheless, farmers noticed lower marmot abundance in 2022 compared to previous years (personal communication), and also snow quantities, known to affect marmot vital rates in other areas of the Alps (Rézouki et al. 2016; Tafani et al. 2013), declined over time. Hence, a population decline in the Dischma Valley within the last 40 years might be in principle possible, however, repeated surveys would be essential for accurately assessing marmot occurrence patterns and changes in marmot absolute numbers in relation to environmental conditions.

## 5 Conclusion

We found no indication of changes in the upper and lower range margins of the Alpine Marmot during the last 40 years of climate change in our study area. However, we detected an increase in the marmot's elevational optimum by +86 m. Our findings indicate that whilst temperature increases may play a role in shifting the marmot's elevational optimum, other environmental factors, such as habitat characteristics and biotic interactions, are equally critical in shaping the species' distribution. The lack of a significant upward shift in range margins suggests that marmots in our study are potentially more constrained by factors other than temperature alone, such as soil conditions, snow cover and vegetation composition. These results underscore the importance of a multifaceted approach to understanding and predicting the impacts of climate change on alpine species. Future research should integrate these factors to better anticipate how marmots and other alpine organisms will adapt to ongoing environmental changes. Moreover, our study highlights the necessity of continuous, long‐term monitoring to accurately assess the effects of climate change on species' distributions and population dynamics.

## Author Contributions

**Miriam Simma:** formal analysis (lead), investigation (lead), methodology (equal), writing – original draft (lead). **Arpat Ozgul:** formal analysis (supporting), methodology (equal), supervision (equal), writing – original draft (supporting), writing – review and editing (supporting). **Francois Duchenne:** formal analysis (supporting), visualization (supporting), writing – review and editing (supporting). **Guido Ackermann:** investigation (equal), writing – review and editing (supporting). **Hannes Jenny:** investigation (equal), writing – review and editing (supporting). **Juerg Paul Müller:** investigation (supporting), writing – review and editing (supporting). **Anne Kempel:** conceptualization (lead), formal analysis (supporting), methodology (equal), project administration (lead), supervision (equal), writing – original draft (supporting), writing – review and editing (supporting).

## Conflicts of Interest

The authors declare no conflicts of interest.

## Supporting information


Data S1:

## Data Availability

The data that support the findings of this study are openly available in Dryad at http://doi.org/10.5061/dryad.hmgqnk9t9.
