# Supplementary material for: Shifting Heights? A 40‐Year Resurvey of Alpine Marmot Distribution in Response to Climate Change
Source: Ecol Evol. 2025 Jul 20;15(7):e71777. doi: 10.1002/ece3.71777 (PMC12277047; doi:10.1002/ece3.71777)
Supplement: Supplementary file 1 — Data S1: [file ECE3-15-e71777-s001.docx]

# Supporting information

**Table S1:** Ranking of candidate models by ΔAIC. The selected, most parsimonious model out of the best-approximating models is highlighted in bold. nPar = number of parameters; ΔAIC = AIC value difference of each model compared to the first ranked model; AICwt = model weight.

| **initial occupancy probability** | **colonisation probability** | **extinction probability** | **detection probability** | **nPar** | **ΔAIC** | **AICwt** |
| --- | --- | --- | --- | --- | --- | --- |
| elevation + elevation^2^ + northness + habitat + slope | elevation + elevation^2^ | elevation + elevation^2^ | distance | 17 | 0 | 0.321 |
| elevation + elevation^2^ + northness + habitat | elevation + elevation^2^ + northness | elevation + elevation^2^ + northness | distance | 18 | 0.54 | 0.245 |
| **elevation + elevation^2^ + northness + habitat** | **elevation + elevation^2^** | **elevation + elevation^2^** | **distance** | **16** | **0.937** | **0.201** |
| elevation + elevation^2^ + northness + habitat + slope + slope*northness | elevation + elevation^2^ | elevation + elevation^2^ | distance | 18 | 1.609 | 0.143 |
| elevation + elevation^2^ + northness + habitat | elevation + elevation^2^ + northness + northness*elevation + northness*elevation^2^ | elevation + elevation^2^ + northness + northness*elevation + northness*elevation^2^ | distance | 22 | 2.561 | 0.089 |
| elevation + elevation^2^ + northness | elevation + elevation^2^ | elevation + elevation^2^ | distance | 12 | 12.705 | 0.001 |
| elevation + elevation^2^ + northness + northness*elevation + northness*elevation^2^ | elevation + elevation^2^ | elevation + elevation^2^ | distance | 14 | 17.272 | < 0.001 |
| elevation + elevation^2^ + northness | elevation | elevation | distance | 10 | 17.322 | < 0.001 |
| elevation + elevation^2^ + northness + habitat | elevation | elevation | distance | 14 | 26.124 | < 0.001 |
| elevation + elevation^2^ + northness + habitat + slope | elevation | elevation | distance | 15 | 28.065 | < 0.001 |
| elevation + northness | elevation | elevation | distance | 9 | 32.092 | < 0.001 |
| elevation | elevation | elevation | distance | 8 | 32.152 | < 0.001 |
| 1 | 1 | 1 | distance | 5 | 47.253 | < 0.001 |
| 1 | elevation | elevation | distance | 7 | 50.174 | < 0.001 |
| 1 | 1 | 1 | 1 | 4 | 246.378 | < 0.001 |


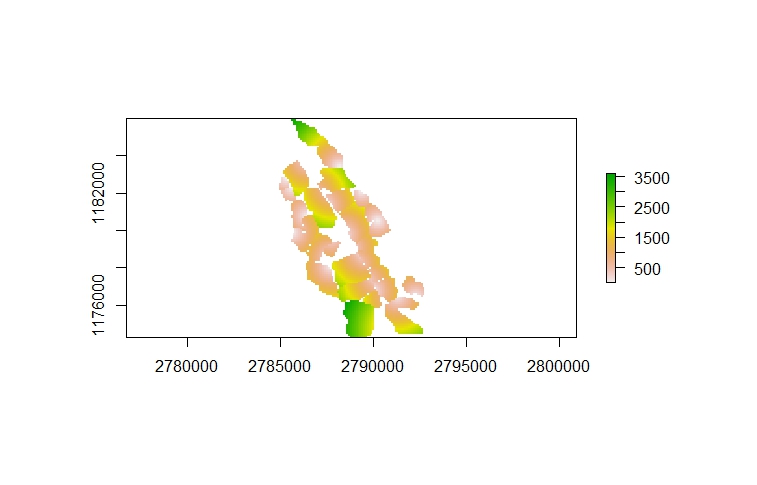


**Figure S1:** Survey distances calculated for each 100x100 m site. The values represent the linear distance (m) between the centre of the grid cell and the corresponding viewpoint (here observation area nr. 16 is not represented because of its overlap with nr. 15). Swiss coordinates (m) for east on the x-axis and for north on the y-axis.


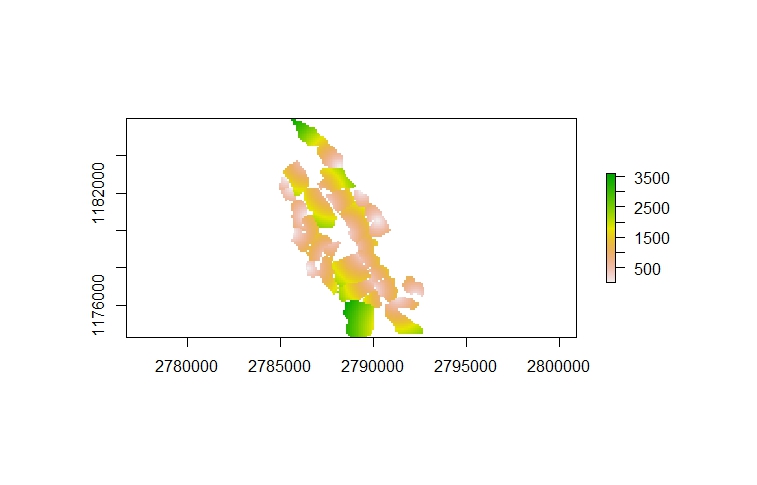


**Figure S2:** Survey distances calculated for each 100x100 m site. The values represent the linear distance (m) between the centre of the grid cell and the corresponding viewpoint (here observation area nr. 15 is not represented because of its overlap with nr. 16). Swiss coordinates (m) for east on the x-axis and for north on the y-axis.


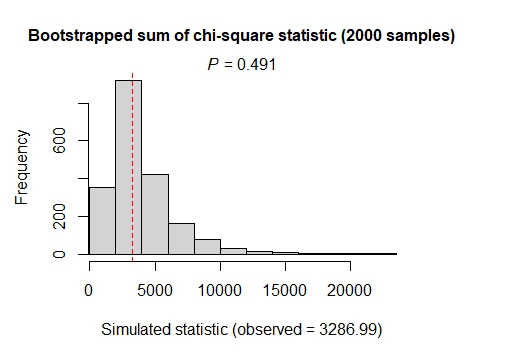


**Figure S3:** Bootstrap statistic derived from the Mackenzie-Bailey Goodness-of-Fit test (MacKenzie & Bailey, 2004) for the selected occupancy model.


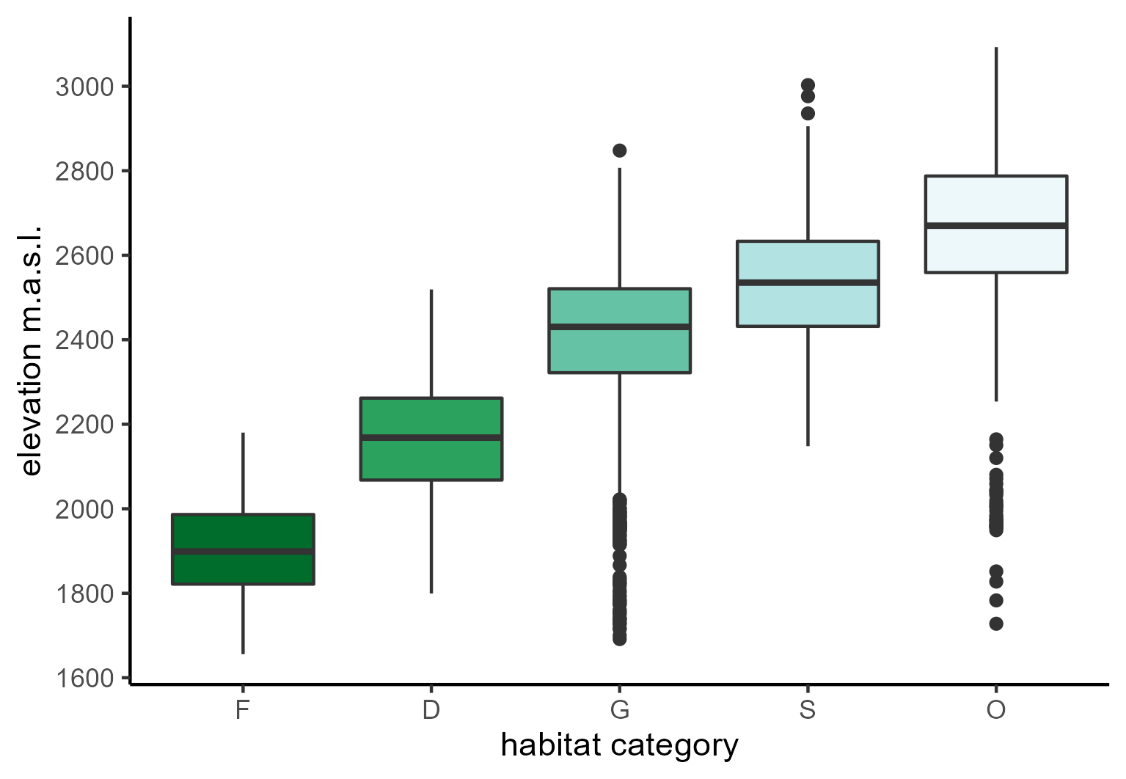


**Figure S4:** Occurrences of habitat categories relative to elevation (F = forest, D = dwarf-shrubs, G = grassland, S = scree, O = other).

**Figure S5**: Number of hunted marmots per year in the Canton of Grison and the Region of Davos. Dotted lines indicate the linear trend. Data obtained from the Cantonal office for hunting and fishing in Grison (Amt für Jagd und Fischerei, Graubünden).


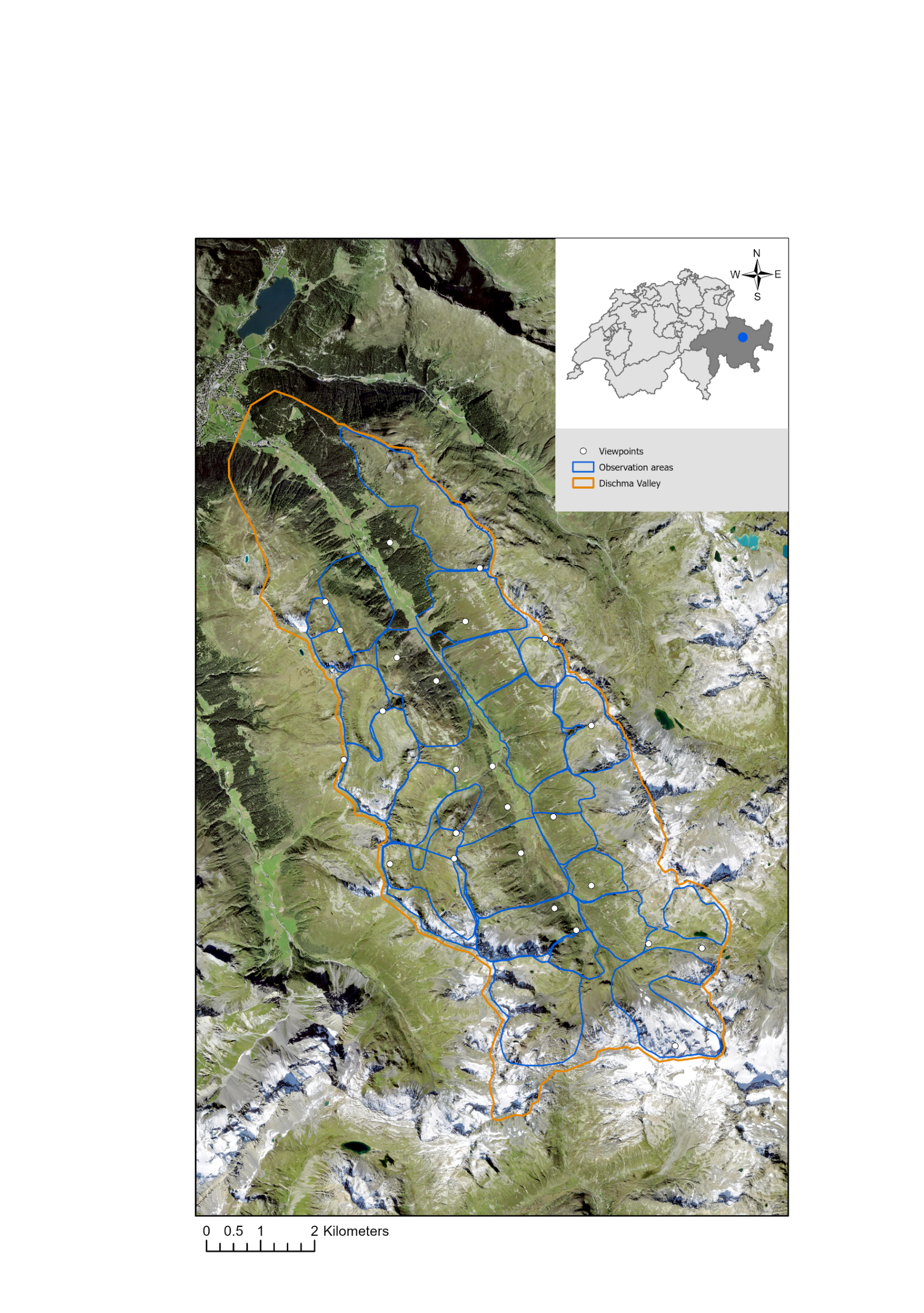


**Figure S6:** Observation areas and viewpoints in the Dischma Valley, canton Grisons, Switzerland. Orthophoto provided by swisstopo.
